# Cyanidin 3-*O*-arabinoside suppresses DHT-induced dermal papilla cell senescence by modulating p38-dependent ER-mitochondria contacts

**DOI:** 10.1186/s12929-022-00800-7

**Published:** 2022-03-07

**Authors:** Young Hyun Jung, Chang Woo Chae, Gee Euhn Choi, Him Cha Shin, Jae Ryong Lim, Han Seung Chang, Joonmo Park, Ji Hyeon Cho, Mo Ran Park, Hyun Jik Lee, Ho Jae Han

**Affiliations:** 1 Department of Veterinary Physiology, College of Veterinary Medicine, Research Institute for Veterinary Science, and BK21 Four Future Veterinary Medicine Leading Education & Research Center, Seoul National University, Seoul, 08826 South Korea; 2 Laboratory of Veterinary Physiology, College of Veterinary Medicine, Chungbuk National University, Cheongju, Chungbuk 28644 South Korea; 3 Institute for Stem Cell & Regenerative Medicine (ISCRM), Chungbuk National University, Cheongju, Chungbuk 28644 South Korea; 4 Biomedical Research Institute, Stempoint Co., Ltd., Seoul, 08501 South Korea

**Keywords:** Cyanidin 3-*O*-arabinoside, DHT, Androgen receptor, Mitochondrial calcium, Senescence

## Abstract

**Background:**

Androgenetic alopecia (AGA) is a genetic disorder caused by dihydrotestosterone (DHT), accompanied by the senescence of androgen-sensitive dermal papilla cells (DPCs) located in the base of hair follicles. DHT causes DPC senescence in AGA through mitochondrial dysfunction. However, the mechanism of this pathogenesis remains unknown. In this study, we investigated the protective role of cyanidins on DHT-induced mitochondrial dysfunction and DPC senescence and the regulatory mechanism involved.

**Methods:**

DPCs were used to investigate the effect of DHT on mitochondrial dysfunction with MitoSOX and Rhod-2 staining. Senescence-associated β-galactosidase activity assay was performed to examine the involvement of membrane AR-mediated signaling in DHT-induced DPC senescence. AGA mice model was used to study the cyanidins on DHT-induced hair growth deceleration.

**Results:**

Cyanidin 3-*O*-arabinoside (C3A) effectively decreased DHT-induced mtROS accumulation in DPCs, and C3A reversed the DHT-induced DPC senescence. Excessive mitochondrial calcium accumulation was blocked by C3A. C3A inhibited p38-mediated voltage-dependent anion channel 1 (VDAC1) expression that contributes to mitochondria-associated ER membrane (MAM) formation and transfer of calcium via VDAC1–IP3R1 interactions. DHT-induced MAM formation resulted in increase of DPC senescence. In AGA mice models, C3A restored DHT-induced hair growth deceleration, which activated hair follicle stem cell proliferation.

**Conclusions:**

C3A is a promising natural compound for AGA treatments against DHT-induced DPC senescence through reduction of MAM formation and mitochondrial dysfunction.

**Supplementary Information:**

The online version contains supplementary material available at 10.1186/s12929-022-00800-7.

## Background

Androgenetic alopecia (AGA) is the most common type of hair loss caused by irregular hair growth cycles. The overactivity of dihydrotestosterone (DHT) produced by 5-α-reductase in human hair dermal papilla cells (DPCs) has been implicated as the main cause of AGA [1]. DHT has the highest affinity for androgen receptor (AR), which is approximately 5 to 10 times greater than that of testosterone. Then, AR bound DHT upregulates the expression of hair growth inhibiting factors such as dikkopf-related protein 1 (DKK-1), interleukin-6 (IL-6), and transforming growth factor β (TGF-β), thereby promoting regression of hair follicles [2, 3]. These secretory factors from senescent DPCs induce hair loss by blocking the transition from telogen (resting phase) to anagen (growth phase) through inhibition of follicle neogenesis, differentiation, and growth of hair follicle stem cells [4]. Reducing DHT-induced DPC senescence is important for AGA prevention and previous researchers strongly agree that mitochondrial dysfunction is a crucial factor in inducing senescence in cells exposed to high DHT concentrations [5–7]. Since therapeutics regulating DHT production by 5-α-reductase inhibition does not have effect on reducing mitochondrial dysfunction and long-term usage of such treatments often brings side effects such as sexual dysfunction or trichosis, it is worthwhile to develop a new therapeutic strategy with reduced side effects while also maintaining the physiological function of mitochondria in DPCs [8–10]. Therefore, understanding the mechanism of DHT-induced mitochondrial dysfunction provides a promising strategy for new AGA treatments that minimize senescence of DPCs exposed to elevated DHT levels.

Mitochondrial dysfunction occurs when formation of mitochondria-associated endoplasmic reticulum (ER) membrane (MAM) increases. This junction impacts cellular metabolism through the regulation of calcium transport between the two structures [11, 12]. Previous research has shown that mitochondrial calcium overload due to increased interaction between inositol 1,4,5-triphosphate receptor (IP3R) and voltage-dependent anion-selective channel protein (VDAC) reduces mitochondrial membrane potential. This, in turn, promotes excessive mitochondrial ROS (mtROS) production leading to mitochondrial dysfunction [13]. Although premature senescence of DPC is tightly associated with excessive mtROS accumulation beyond its physiological antioxidative capacities, the involvement of MAM formation in the process of DPC senescence is unclear. Previous studies, including our own, have shown that dysregulation of MAM formation leads to cellular function changes in calcium homeostasis, ROS production, autophagy, and lipid metabolism [14]. Influx of calcium to the mitochondrial matrix through IP3R–VDAC1 coupling affects mitochondrial membrane potential, mitochondrial respiration, and mitochondrial permeability transition pore (mPTP) formation, all leading to mitochondrial dysfunction [13, 15]. Moreover, MAM-dependent mitochondrial calcium accumulation mediated by IP3R–VDAC1 is a critical factor in cellular senescence and aging [12, 16]. Taken together, this illustrates the need to investigate the mechanism inducing MAM-dependent mitochondrial calcium accumulation in response to DHT-mediated signal transductions for reducing DPC senescence.

Cyanidins are a major flavonoid anthocyanin. They are the main pigments in various fruits, flowers, and leaves. They are powerful antioxidants having therapeutic potential in anti-aging, anti-cancer, anti-inflammation, neuroprotection, and cardioprotection [17, 18]. Owing to their oxygen radical scavenging capacity, many researchers investigate the effects of cyanidins on preventing the aging process. Furthermore, due to low side effects and toxicities of cyanidins, derivative effects of scavenging ROS by modulating signal transduction pathways have gained interests of researchers [19, 20]. The bioactive properties of cyanidins are associated with the regulation of mitochondrial functions [21]. In addition, cyanidins have protective effects on various diseases by regulation of mitochondrial copy number, mitochondrial respiratory chain, mitochondrial membrane potential, and mitophagy [22–25]. However, the effects of cyanidins on mitochondrial functions can vary depending on the attached sugar residues. Although the protective effects of cyanidins on mitochondrial dysfunction have been studied, the method in which cyanidins modulate DHT-mediated mitochondrial dysfunction and DPC senescence has not. Therefore, finding promising candidates for using cyanidins to prevent DPC senescence will be helpful for AGA treatment. In this study, we investigated the effect of cyanidins on DHT-mediated DPC senescence and the detailed mechanisms related to regulation of mitochondrial calcium homeostasis. We also aimed to identify the protective effect of cyanidins on DPC senescence-mediated hair loss in an AGA mice model.

## Methods

### Materials

Cyanidin 3-*O*-arabinoside (C3A, #26183) and cyanidin 3-*O*-glucoside (C3-glu, #16406) were obtained from Cayman (Ann Arbor, MI, USA). Cyanidin 3-*O*-galactoside was purchased from PhytoLab (Vestenbergsgreuth, Germany, #PHL89463-10MG). 5α-Dihydrotestosterone (DHT) solution (Cerilliant, Round Rock, TX, USA, #D-073) and 5α-ANDROSTAN-17β-OL-3-ONE 3-O-CARBOXYMETHYLOXIME: BSA (Steraloids, Wilton, NH, USA, #A2574-050) were acquired. MitoTEMPO (#ALX-430-150-M005) was purchased from Enzo life science (Farmingdale, NY, USA). H2DCFDA (#D399), MitoSOX (#M36008), DAPI (#D1306), Bovine serum albumin (BSA, #15561020), ER-Tracker Red (#E34250), Mito-Tracker Green FM (#M7514), and Rhod-2 AM (#R1244) were obtained from Invitrogen (Carlsbad, CA, USA). Apocynin (#178385), SB203580 (#S8307), Bicalutamide (#PHR1678) and Castor oil (#259853) were acquired from Sigma-Aldrich (Sternheim, Germany). TurboFect (#R0532) was purchased from Themo Scientific (Waltham, MA, USA). Ki67 (#ab16667), IP3R1 (#ab5804), and p16INK4a (#ab108349) antibodies were acquired from Abcam (Cambridge, UK). AR (#51135), p38 (#9212S), phospho-HSP27 (#2401L), and MCU (#14997S) antibodies were purchased from Cell signaling (Danvers, MA, USA). HSP27 (#MA3-015) and p21 (#MA5-14949) antibodies were obtained from Invitrogen. Phospho-p38 (#SC-166182), phospho-ERK (#SC-7383), phospho-JNK (#SC-6254), ERK-1 (#SC-94), JNK (#SC-7354), Lamin A/C (#SC-20681), VDAC1 (#SC-390996), and β-actin (#SC-47778) antibodies were bought from Santa Cruz (Dallas, TX, USA). α-tubulin (#T6074) antibody was obtained from (Sigma-Aldrich). To avoid the oxidation of chemicals, all materials were prepared fresh at the moment of use. All reagents were of the highest purity available.

### Cell culture

DPCs were cultured in hair follicle dermal papilla cell growth medium (containing supplement) provided by Promocell, which was supplemented with a 1% penicillin–streptomycin solution (Gibco, Grand Island, NY, USA). We used DPCs both expressing AR and responding to DHT. Cells were grown in 60 mm dishes, 100 mm culture dishes, or a 96-well plate (Corning, NY, USA) in an incubator (37 °C, CO_2_ 5%, and air 95%). When the cells reached 80% confluency, the culture medium was replaced with supplement-free medium for 24 h for starvation. After 24 h, the cells were incubated in supplement-free medium with the indicated agents for the designated treatment time. Human hair follicle stem cells (HFSCs) (Celprogen, Torrance, CA, USA) were acquired and used in this study. HFSCs were cultured with human hair follicle maintenance medium (HFSC media, Celprogen) provided with supplement. HFSCs were cultured in human hair follicle expansion extracellular matrix coated flasks (Celprogen). When HFSCs reached 80% confluency, the media was replaced with a mixture of DPC-conditioned media and HFSC media in a ratio of 1:1 and cultured for 48 h. Viable cells were counted with trypan blue exclusion test.

### Measurement of intracellular ROS, mtROS and mitochondrial calcium

The level of mitochondrial superoxide and calcium was measured by Cytoflex (Beckman Coulter, FL, USA), as follows. Trypsinized DPCs were incubated with MitoSOX (5 μM) or Rhod-2 (2 μM) for 20 min at 37 °C while protected from light. Cells were washed once with phosphate-buffered saline (PBS), resuspended in PBS, and analyzed by flow cytometry. The results were obtained by comparing the percentage of cells with high fluorescence intensity. The intracellular level of ROS was measured with H2DCFDA staining. Cells were washed with PBS and incubated in 1 μM of H2DCFDA in culture media at 37 °C for 30 min. Trypsinized cells were analyzed by Cytoflex.

### Senescence-associated β-galactosidase activity assay

DPCs were treated following the experimental design and washed twice with PBS. Cells were fixed with 4% paraformaldehyde (PFA, Lugen Sci, Bucheon, Korea) for 10 min at room temperature and then incubated with senescence-associated β-galactosidase (SA-β-gal) (Sigma-Aldrich, #CS0030) staining solution for 12 h at 37 °C without CO_2_. The SA-β-gal-positive cells were observed in blue color by using an inverted microscope (Nikon Eclipse TS100; Nikon, Tokyo, Japan) and the mean percentages of SA-β-gal-positive cells were calculated.

### Growth factor antibody array

Culture conditioned media were analyzed for content of growth factors by incubation with membranes of the RayBiotech C-Series Human Growth Factor Antibody Array C1 kit, AAH-GF-1 (RayBiotech, Norcross, GA, USA). The membranes were incubated in blocking buffer for 30 min, followed by overnight incubation at 4 °C with conditioned media. The membranes were then washed five times with wash buffer and incubated for 2 h with biotin-conjugated antibodies at room temperature. Then, the membranes were washed five times with wash buffer and incubated for 2 h with horseradish peroxidase-conjugated streptavidin. After the washing process, human growth factors were detected by enhanced chemiluminescence reagents using a chemiluminescence imaging system. Analysis was done with relative optical density of the spots and normalized with positive controls. The antibodies used for growth factor antibody array is listed in Additional file 1: Table S1.

### Western blot and subcellular fractionation

Cells were lysed to RIPA buffer with a protease inhibitor cocktail and incubated for 30 min on ice. Cell lysates were downed by centrifugation at 12,000 rpm for 20 min at 4 °C. After spin down, the protein concentration was determined using Pierce BCA Protein Assay Kit (Thermo scientific, Waltham, MA, USA) at 562 nm, and equal amounts of protein (10 μg) were separated by SDS-PAGE (8–12% polyacrylamide). The protein was transferred to polyvinylidene difluoride (PVDF) membranes by semi-dry transfer cells (Bio-Rad, Hercules, CA, USA) at 22 V for 1 h. The membranes were blocked with 5% non-fat skim milk (Blocking-Grade Blocker; Bio-Rad) in 1× TBST (10 mM Tris–HCl, 150 mM NaCl, 0.05% Tween-20) at room temperature for 30 min and incubated with primary antibodies at 4 °C overnight. The membranes were incubated with horseradish peroxidase (HRP) conjugated with anti-IgG secondary antibodies for 2 h and visualized by an ECL Detection Kit (Bio-Rad). The specific bands were visualized using the ChemiDoc™ XRS + System (Bio-Rad). Subcellular fractionation was conducted to isolate cytosolic and nuclear proteins. Cells were cultured in 100 mm dishes and treated with the indicated reagents. For the preparation of cytosolic- and nuclear-fractionated samples, the EzSubcell subcellular fractionation/extraction kit (Atto, Tokyo, Japan, #WSE-7421) was used. Cytosolic and nuclear samples for western blot analysis were prepared according to the manufacturer’s instructions. α-tubulin and Lamin A/C were used as cytosolic and nuclear protein markers, respectively.

### AR transcriptional activity assay

DPCs were grown in 96 well black plate and transfected with Cignal Androgen Receptor Reporter (200 ng) with TurboFect transfection reagent (Thermo scientific, #R0532) according to the manufacturer’s instructions (Qiagen, Venlo, Netherlands, #CCS-1019L). Cells were grown to 80% confluency and pretreated with C3A (1 μM) prior to exposure to DHT (100 nM). They were analyzed for Firefly/Renilla luciferase activities in Victor 3 multilabel plate reader (PerkinElmer, Boston, MA, USA) using luciferase reporter assay system (Promega, Madison, WI, USA, #E1910) following the manufacturer’s guideline.

### Real-time quantitative PCR

Cells were treated with DHT or a vehicle for 24 h. Cells were then washed with PBS twice and lysed with buffer RL containing a 50× Dithiothreitol (DTT) solution. Total RNA was extracted using an RNA Extraction kit (Takara, Japan, #9767) according to the manufacturer’s instructions. Reverse-transcription PCR (RT-PCR) was conducted with 0.5 μg of total RNA using a Maxime RT premix kit (iNtRON, Sungnam, Korea). The relative mRNA expression level of the target gene was analyzed using a Rotor-Gene Q device (Corbett Research, Cambridge, UK) with the TB Green Premix Ex Taq (TaKaRa, #RR420A). The specificity, efficiency, and fidelity of PCR primers for real-time quantitative PCR were validated by checking PCR products and analyzing the melting curves. Primer sequences are listed in Additional file 1: Table S2.

### Immunocytochemistry

Cells were cultured in confocal dishes with diameters of 35 mm (SPL, Seoul, Korea) and fixed with 4% PFA in PBS, permeabilized for 5 min with 0.1% (v/v) of Triton X-100, and washed at each step three times with PBS for 5 min. The cells were blocked with 5% normal goat serum (NGS, Vector Lab, Burlingame, CA, USA) in PBS for 30 min. Anti-AR antibody was diluted in 5% (v/v) NGS in PBS for 4 h at RT. After washing with PBS three times for 5 min, the cells were incubated with Alexa Fluor 555 secondary antibodies (Invitrogen) for 1 h at room temperature and counterstained with DAPI (Invitrogen). Cells were visualized with a Andor SRRF system (Oxford instruments, Abingdon, UK). For analyzing ER-mitochondrial contacts, cells were treated with C3A and DHT for 24 h. After washing the cells with PBS three times, cells were incubated with Mito-Tracker green (200 nM) and ER-Tracker (200 nM) in supplement-free medium for 20 min at 37 °C, and nuclei were stained with DAPI.

### Small interfering RNA (siRNA) transfection

DPCs were grown to 80% confluency and the cells were transfected for 24 h with the pre-designed siRNAs for targeting *AR* or *VDAC1* acquired from BIONEER (Daejeon, Korea) or a non-targeting (NT) siRNA as a negative control (25 nM) with the TurboFect reagent (Thermo Scientific), according to the manufacturer’s protocol. The sequences of siRNA used in this study are listed in Additional file 1: Table S2.

### Enzyme-linked immunosorbent assay (ELISA)

For the quantification of DKK-1 in DPC-conditioned medium, the Human DKK-1 Quantikine ELISA Kit (R&D systems, Minneapolis, MN, USA, #DKK100B) was used, according to the supplier’s protocol. DPCs were cultured to 80% confluency and then treated with DHT. Conditioned media was acquired and preserved in − 80 °C. After centrifugation at 3000 *g* for 5 min, supernatants were used for the ELISA assay. IL-6 and TGF-β1 concentrations in DPC-conditioned medium were measured with human IL-6 (LabisKOMA, Seoul, Korea, #K0331194) and TGF-β1 (LabisKOMA, Seoul, Korea, #K0332110) ELISA kits.

### In situ proximity ligation assay

VDAC1/IP3R1 interactions were detected in situ using Duolink^®^ In Situ Red Starter Kit Mouse/Rabbit (Sigma-Aldrich, # DUO92101) according to the supplier’s protocols. Cells were fixed and proximal ligation assay (PLA) probe, anti-VDAC1 and anti-IP3R1 antibodies were applied. Then, secondary antibodies were added. If the antibodies were in close proximity (< 40 nm), they ligated together. Polymerization and amplification solutions were supplemented to amplify the signal (red) of the closed circle and were visualized by SRRF microscopy. DAPI was used to counterstain the nuclei.

### Experimental design of the animal study

All animal experiments were conducted in accordance with the National Institutes of Health Guide for the Care and Use of Laboratory Animals and permitted by the Institutional Animal Care and Use Committee of Seoul National University (SNU-200916-6-1). Seven-week-old male C57BL/6 mice (ORIENT, Seongnam, Korea) adapted for 1 week were administered DHT (1 mg/day) dissolved with corn oil, days -1, 5, and 13, intraperitoneally. At the day after first DHT injection, all mice were anesthetized by intraperitoneal injection of alfaxalone (40 mg/kg) with xylazine (10 mg/kg). The mice were shaved with clippers and depilated with hair removal cream. The anagen phase was induced in the dorsal skin of the mice. The mice were divided into five groups. C3A (500 μM), Apocynin (100 mM), and SB203580 (1 mM) were prepared and dissolved in a mixture of castrol oil (Sigma-Aldrich, #259853) and ethanol in a 7:3 ratio. They were treated topically on the depilated skin once every 2 days. Mice of dorsal skin and hair growth states were captured with a digital camera at the distance of 20 cm at days 7, 11, 15 and 19. At day 21, all mice were sacrificed for acquiring skin tissue samples.

### Hematoxylin and eosin (HE) staining and immunohistochemistry

To evaluate skin thickness and hair regrowth, skin samples were collected 21 days after depilation. Samples were fixed in 4% PFA for 24 h and then dehydrated in 30% sucrose dissolved in PBS for 1 day. Longitudinal 30-μm-thick sections were obtained using a cryostat (Leica Biosystems, Nussloch, Germany) and then stained with HE. The tissues were incubated with 4% PFA for 5 min and washed with running tap water for 5 min. The tissues were stained with hematoxylin for 3 min and were rinsed with running tap water for 5 min. Next, the tissues were incubated with 70% alcohol containing 1% HCl for 5 s and incubated with eosin solution for 30 s. The slides were placed in 95%, 100% ethanol, and xylene three times, each for 3 s. The tissues were covered with a thin coverslip (Paul Marienfeld GmbH & Co. KG, Lauda-Konigshofen, Germany). Skin thickness and diameter of dermal papilla were measured using Slideviewer software (3DHISTECH, Budapest, Hungary). The dermal ratio was measured using the ratio of the thickness between the epidermis and the dermis to the entire skin thickness. Immunohistochemistry was conducted with anti-Ki67 antibody. The nuclei were stained with DAPI. Immunofluorescence image was captured by confocal microscopy (Carl Zeiss, Oberkochen, Germany, #LSM 800). Histological evaluations were performed in a blind fashion.

### Statistical analysis

All quantitative data are presented as the mean ± standard error of the mean (SEM). Data were analyzed using SigmaPlot 12 software. The sample sizes for animal studies were determined by SigmaPlot 12 software. Comparisons between two experimental groups were performed using a two-tailed Student’s t-test. The means of multiple experimental groups were compared using a one-way ANOVA, followed by the Student–Newman–Keuls test for multiple comparisons. A p-value of < 0.05 was considered statistically significant.

## Results

### Effect of C3A on DHT-induced DPC senescence

**Fig. 1:**
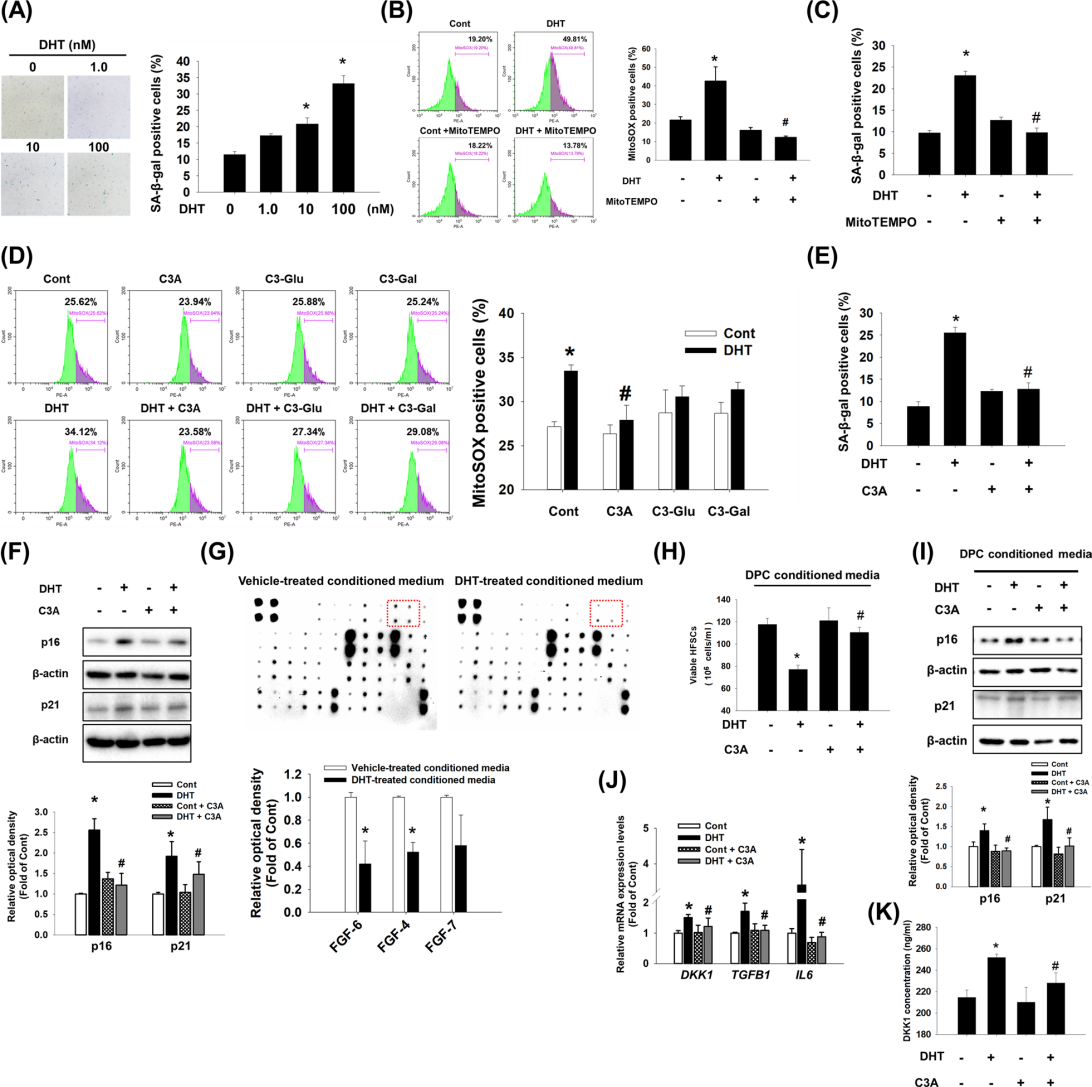
Effect of C3A on DHT-induced DPC senescence. **A** Dermal papilla cells (DPC) were treated with dihydrotestosterone (DHT) (0 to 100 nM) for 72 h. Senescent cells were determined by senescence associated β-galactosidase activity (SA-β-gal) assay. The positive cells (blue) were counted in five random fields manually. Proportional number of positive cells was presented by the percentage of cells of each treat group. N = 5. **B** Cells were pretreated with MitoTEMPO (1 μM) for 30 min and treated with DHT (100 nM) for 48 h. Cells were stained with MitoSOX and analyzed by flow cytometer. N = 4. **C** Cells were pretreated with MitoTEMPO (1 μM) for 30 min and treated with DHT (100 nM) for 72 h. Senescent cells were detected with SA-β-gal assay. N = 5. **D** Cells were pretreated with cyanidin 3-*O*-arabinoside (C3A) (1 μM), cyanidin-3-*O*-glucoside (C3-glu, 1 μM), and cyanidin-3-*O*-galactoside (C3-gal, 1 μM) for 30 min and treated with DHT for 48 h. Cells were stained with MitoSOX and analyzed by flow cytometer. N = 4. **E** Cells were pretreated with C3A for 30 min and exposed to DHT for 72 h. SA-β-gal assay was performed. N = 5. **F** Cells were pretreated with C3A for 30 min and treated DHT for 72 h. The protein expression levels of p16 and p21 were analyzed by western blot analysis. β-actin was used as a loading control. N = 4. **G** DPCs were treated with vehicle or DHT for 72 h. DPC conditioned media was used for growth factor antibody array. The results were captured by chemiluminescence imaging system and quantified by relative optical densities of spots. FGF-6, FGF-4, and FGF-7 expression levels were compared with control. N = 3. **H**, **I** DPC conditioned media were obtained from the culture media of DPCs pretreated with C3A and treated with DHT for 72 h. Human hair follicle stem cells (HFSCs) were treated with DPC conditioned media for 48 h. **H** Viable cells were counted with trypan blue exclusion assay. N = 6. Data are shown by cell numbers in 1 ml. **I** Protein expression levels of p16 and p21 were evaluated by western blot analysis. N = 4. **J** DPCs were treated with C3A for 30 min prior to DHT exposure for 24 h. The mRNA expression levels of *DKK1*, *TGFB1*, and *IL6* analyzed by using qPCR. N = 5. **K** DKK-1 concentration in the DPC conditioned media by using DKK-1 ELISA kit. DPC conditioned media was taken from the culture media of DPCs pretreated with C3A and treated with DHT for 72 h. N = 5. Data are mean ± SEM. *p < 0.05 versus Control. ^#^p < 0.05 versus DHT

DHT is widely known to induce DPC senescence. We performed a SA-β-gal activity assay, which reflects the state of aging, to investigate the effect of DHT on DPC senescence. We confirmed that the ratio of SA-β-gal-positive DPCs was increased by DHT in a concentration-dependent manner (Fig. 1A). A senescent cell is characterized by increased mtROS levels, so we examined the mtROS levels in DHT-treated DPCs. We found that DHT-induced mtROS accumulation was reduced by MitoTEMPO, mitochondria-targeted antioxidant, which was evaluated by MitoSOX staining. MitoTEMPO recovered DHT-induced DPC senescence (Fig. 1B, C). We hypothesized that cyanidins could influence mtROS levels in DHT-induced DPC senescence. Among cyanidin candidates, the mtROS reducing effect of C3A was higher than C3-glucoside (C3-Glu) or C3-galactoside (C3-Gal) (Fig. 1D). Indeed, we observed that C3A pretreatment reduced the ratio of SA-β-gal-positive DPCs that had increased with DHT treatment (Fig. 1E). Consistently, C3A pretreatment inhibited the expression of senescence markers p21 and p16 that increased with DHT in DPCs (Fig. 1F). Given that DPC senescence modifies secretion of various growth factors that support HFSCs proliferation, we determined the levels of growth factors after treating with DHT. In antibody arrays detecting growth factors, decreased FGF6 and FGF4 were observed in DHT-conditioned media (Fig. 1G). To confirm whether C3A-treated DPC-conditioned media suppresses the deleterious effect of DHT-treated DPC-conditioned media on HFSC proliferation, HFSCs were cultured with DPC-conditioned media treated with DHT or C3A. We found that DPC-conditioned media treated with DHT reduced proliferation of HFSCs but C3A pretreatment reduced the deleterious effect of DHT (Fig. 1H). The secreted factors from senescent DPCs inhibits HFSC proliferation. However, C3A recovered the HFSC proliferation when exposed to DHT-treated conditioned media. We found that HFSCs exposed with C3A-pretreated DPC-conditioned media have shown decreased levels of senescence marker expression compared to those of HFSCs exposed to DHT-treated DPC-conditioned media (Fig. 1I). Furthermore, we detected the mRNA levels of *DKK1*, *TGFB1*, and *IL6*, which are known to promote hair follicle regression. As expected, increased *DKK1*, *TGFB1*, and *IL6* mRNA levels were reversed by C3A pretreatment (Fig. 1J). Consistent with these results, increased DKK1 concentrations in DHT-treated conditioned media were reduced to control levels with C3A (Fig. 1K). In addition, increased IL-6 and TGF-β1 concentrations in DHT-treated conditioned media were also recovered to control levels with C3A (Additional file 1: Fig. S1). Collectively, we found that C3A effectively reduced DHT-induced DPC senescence and the associated secretory factors. Also, C3A pretreatment rescued the proliferation of HFSCs exposed with DHT-treated conditioned media, which maintains hair follicle regeneration ability.

### Effect of C3A on DHT-induced nuclear translocation of AR

**Fig. 2:**
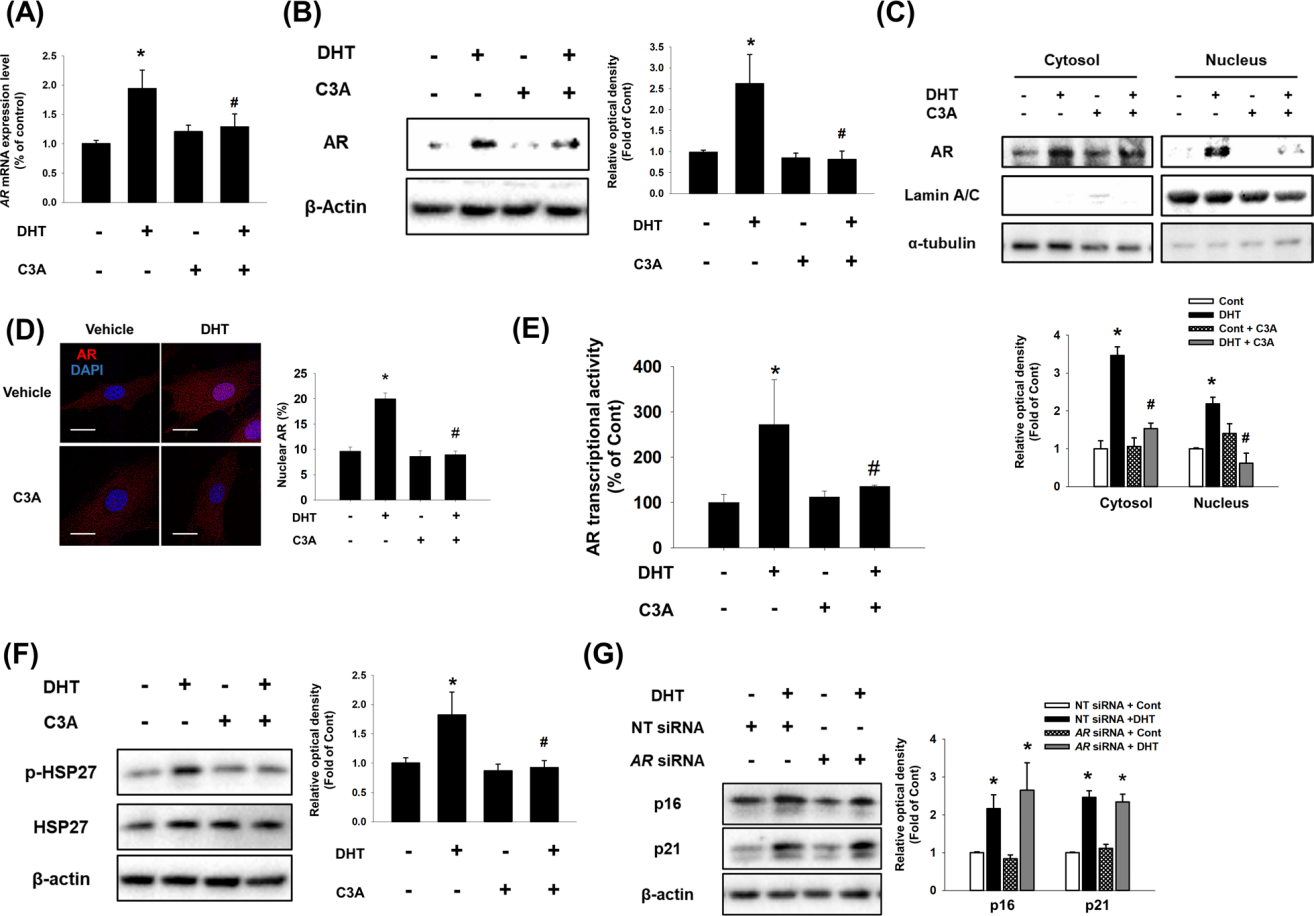
Effect of C3A on DHT-induced nuclear translocation of AR. **A**–**F** DPCs were treated with C3A for 30 min prior to DHT for 24 h. **A** The relative mRNA expression level of *AR* was analyzed by real time PCR. Normalization was achieved by *ACTB mRNA* expression level (**B**) AR protein expression level was detected by western blot analysis. N = 3. **C** Nuclear and cytosolic proteins were separated by intracellular fractionation. Protein expression level of AR was detected. Lamin A/C was used as a nuclear marker, and α-tubulin was used as a cytosol marker. N = 3. **D** Cells were immunostained with anti-AR antibody (red). Nucleus is counterstained with DAPI (blue). N = 3. Magnification ×1000. Scale bars are 8 μm. **E** AR transcriptional activity was analyzed by Cignal reporter assay. N = 5. **F** Phosphorylated HSP27 and HSP27 protein expression levels were analyzed by western blotting. N = 3. **G** Cells were transfected with non-targeting (NT) siRNA (25 nM) and *AR* siRNA (25 nM) and treated with DHT for 72 h. Western blotting analysis was performed for p16 and p21 proteins. β-actin was used as a loading control. N = 3. Data are mean ± SEM. *p < 0.05 versus Control. ^#^p < 0.05 versus DHT

Binding of androgens to the AR induces nuclear translocation of AR, which then induces AR expression and acts as a transcription factor for androgen-responsive genes [26, 27]. Thus, we measured *AR* mRNA expression and AR protein expression in DPCs to determine whether C3A affects DHT-induced AR expression. DHT increased *AR* mRNA and AR protein expression, but their expressions were reduced to control levels with C3A treatment (Fig. 2A and B). Next, we investigated the effect of C3A on the nuclear translocation of AR. DHT-induced AR nuclear translocation and C3A pretreatment suppressed the translocation of AR into the nucleus (Fig. 2C). Similarly, we observed that increased AR fluorescence intensity by DHT treatment in the nucleus was reversed by C3A (Fig. 2D). Furthermore, we investigated AR transcription activity through luciferase reporter assay in DPCs. AR transcription activity was increased by about 250% with DHT, but significantly decreased to control levels with C3A pretreatment (Fig. 2E). DHT-bound AR interacts with heat shock protein 27 (HSP27). When phosphorylated HSP27 is bound with AR, it translocates into the nucleus [28, 29]. Therefore, we assumed that C3A would inhibit AR nuclear translocation by affecting the interaction between AR and HSP27. We found that DHT increased the phosphorylation of HSP27, which was decreased with C3A pretreatment (Fig. 2F). Since cytoplasmic AR is completely different from the membrane ARs in terms of gene locus and molecular structure, we then used *AR* siRNAs specific for cytoplasmic AR but not mAR to investigate whether the nuclear translocation of AR is involved in DPC senescence. Interestingly, silencing AR expression did not suppress DHT-induced expression of p16 and p21 (Fig. 2G). Collectively, C3A plays a key role in inhibiting DHT-induced AR nuclear translocation through the regulation of HSP27 phosphorylation, but nuclear translocation of AR is not involved in DHT-induced DPC senescence.

### Effect of C3A on DHT-induced p38 phosphorylation

**Fig. 3:**
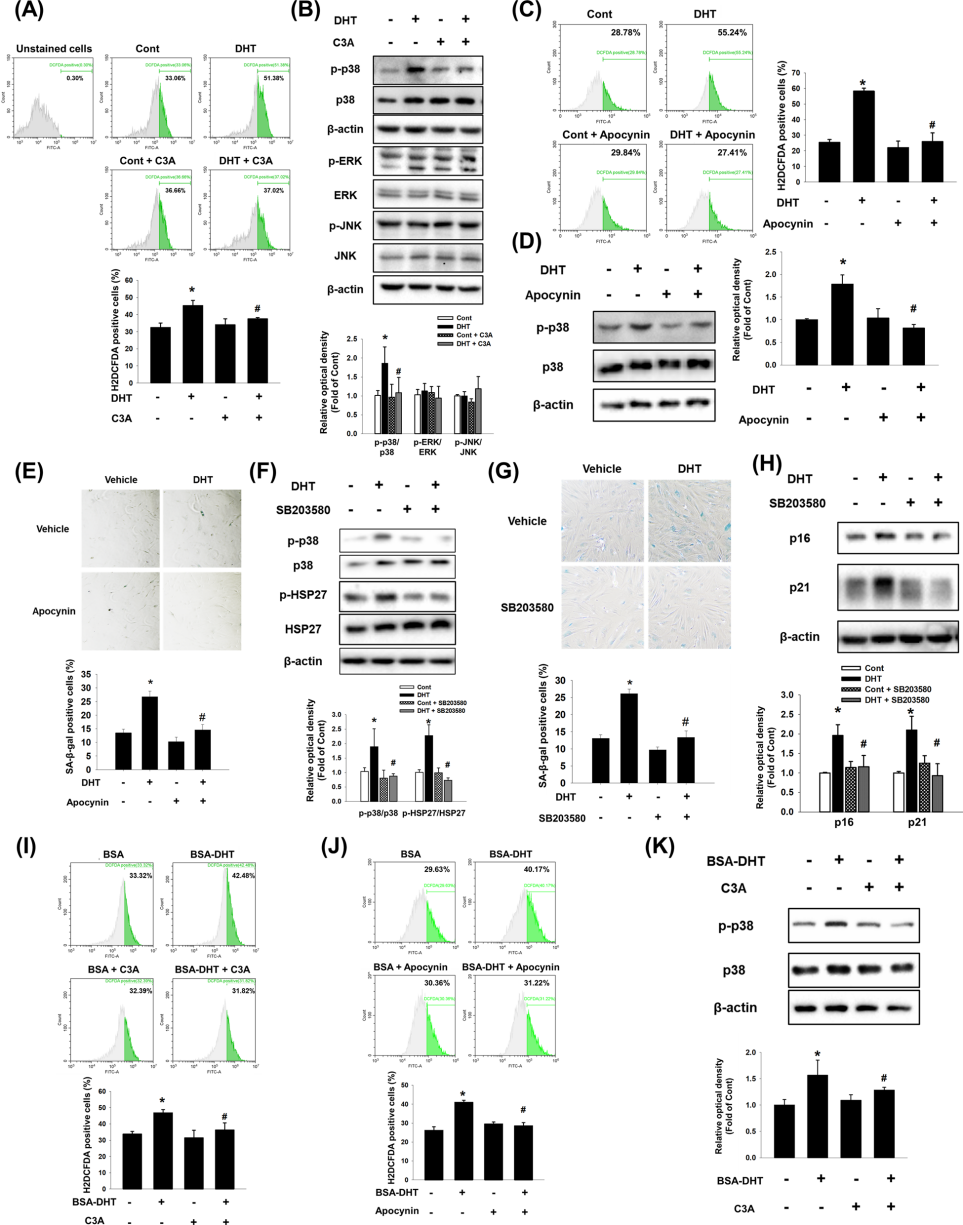
Effect of C3A on DHT-induced p38 phosphorylation. **A**, **B** DPCs were treated with C3A for 30 min and treated with DHT for 2 h. **A** Cells were stained with H2DCFDA (1 μM) and analyzed with flow cytometer. N = 3. **B** The levels of p-p38, p-ERK, and p-JNK were determined by western blot analysis. Data are normalized by each anti-p38, -ERK and -JNK antibodies. N = 3. **C**, **D** Cells were treated with Apocynin (100 μM) for 30 min and exposed to DHT for 2 h. **C** Cells were stained with H2DCFDA and analyzed by flow cytometer. N = 3. **D** Phosphorylated p38 expression level was analyzed by western blot analysis. N = 4. **E** Cells were treated with Apocynin for 30 min and exposed to DHT for 72 h. SA-β-gal activity assay was performed, and blue stained cells of total cells were counted. N = 5. **F** Cells were treated with SB203580 (1 μM) for 30 min and exposed to DHT for 24 h. Phosphorylated p38 and HSP27 expression levels were analyzed by western blot analysis. N = 3. **G**, **H** Cells were treated with SB203580 (1 μM) for 30 min and exposed to DHT for 72 h. **G** SA-β-gal activity assay was performed, and blue stained cells of total cells were counted. N = 5. **H** Western blotting was achieved for analyzing p16 and p21 protein expression levels. N = 3. **I** Cells were treated with C3A for 30 min and exposed to BSA conjugated DHT for 2 h. Cells were stained with H2DCFDA (1 μM) and H2DCFDA-positive cells were analyzed by flow cytometer. N = 6. **J** Cells were treated with Apocynin for 30 min and exposed to BSA conjugated DHT for 2 h and stained with H2DCFDA (1 μM). H2DCFDA-positive cells were analyzed by flow cytometer. N = 3. **K** Phosphorylated p38 expression level was analyzed by western blot. N = 4. Data are mean ± SEM. *p < 0.05 versus Control. ^#^p < 0.05 versus DHT or BSA-DHT

Then, we investigated whether DHT triggers DPC senescence by activating membrane AR (mAR). The mAR is widely known to activate NADPH oxidase (NOX) to produce ROS, which then activates mitogen-activated protein kinase (MAPK) [30]. Therefore, we detected intracellular ROS by H2DCFDA staining. DHT treatment increased ROS, which was reversed by C3A (Fig. 3A). We then investigated which MAPK activity was affected by DHT and C3A. We found that C3A pretreatment reduced the DHT-induced phosphorylation of p38, whereas it did not significantly affect phosphorylation of ERK or JNK (Fig. 3B). Pretreatment with Apocynin, a NOX complex inhibitor, reduced DHT-induced ROS production, demonstrating that DHT increases ROS formation through activating the NOX complex (Fig. 3C). DHT-induced p38 phosphorylation and DPC senescence were inhibited by Apocynin pretreatment (Fig. 3D and E). We then investigated whether the HSP27 phosphorylation by DHT was induced by p38-mediated signaling. Treatment with the p38 inhibitor, SB203580, reduced DHT-induced HSP27 phosphorylation (Fig. 3F), indicating that senescence inhibition by C3A was mediated by decreasing ROS-mediated p38 activity. Furthermore, pretreatment with SB203580 effectively prevented DHT-induced senescence in DPCs, as demonstrated by reduced SA-β-gal activity and the expression of p16 and p21 (Fig. 3G and H). When we performed an experiment by treatment of C3A and SB203580 simultaneously, DHT-induced senescence marker expressions of p16 and p21 was reduced similar to that of C3A treatment group (Additional file 1: Fig. S2). These results suggest that C3A-inhibited DPC senescence is mediated by the suppression of p38 activity induced by DHT. Furthermore, to confirm the involvement of mAR-mediated signaling, we treated membrane impermeable DHT. The mARs are predicted to have transmembrane domains and localized to plasma membrane, and DHT initiates rapid signaling activating mARs. Our results showed that expressions of *OXER1* and *SLC39A* were confirmed but *GPRC6A* and *TRPM8* were not in DPCs (Additional file 1: Fig. S3A). Indeed, *AR* silencing did not reduce both *OXER1* and *SLC39A* mRNA expression levels (Additional file 1: Fig. S3B). Since BSA-conjugated DHT (BSA-DHT) activates mAR rather than intracellular AR, we examined the effects of BSA-DHT on NOX-dependent ROS formation and p38 phosphorylation. BSA-DHT increased ROS levels, which were inhibited by Apocynin and C3A pretreatments (Fig. 3I and J). Subsequently, DHT-BSA-induced p38 phosphorylation was reversed by C3A pretreatment (Fig. 3K). Therefore, our results indicate that DHT-activated mAR induces DPC senescence. In addition, p38-mediated HSP27 phosphorylation is inhibited by C3A via regulation of NOX-dependent ROS formation.

### Effect of C3A on DHT-induced ER-mitochondria contacts

**Fig. 4:**
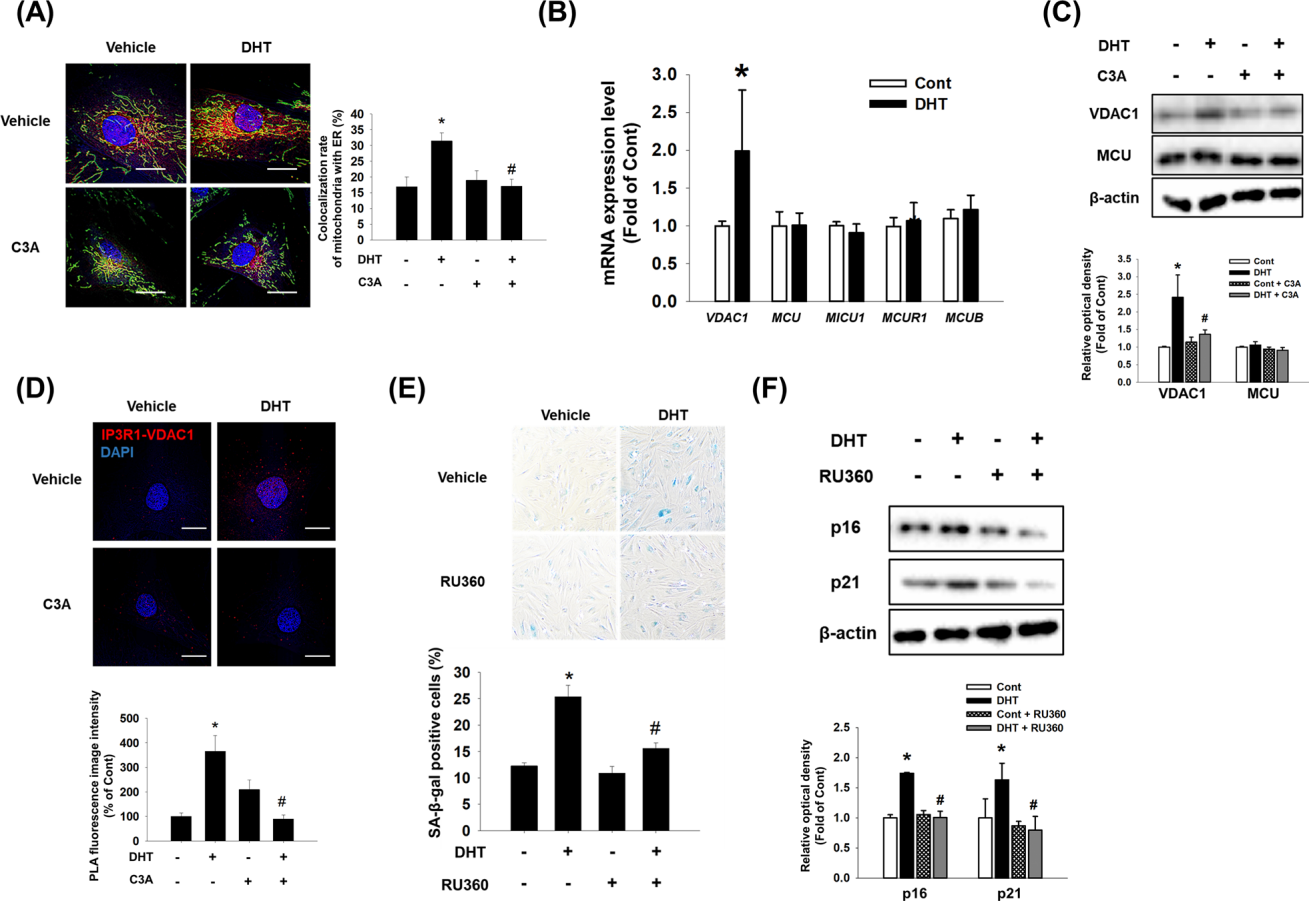
Effect of C3A on DHT-induced ER-mitochondria contacts. **A** DPCs were treated with C3A and exposed to DHT for 24 h. Cells were visualized by staining with ER-Tracker (200 nM) and Mito-Tracker (200 nM). Colocalization of ER-Tracker and Mito-Tracker was analyzed by Image J software. N = 5. Magnification ×1000. Scale bars are 8 μm. **B** Cells were treated with DHT for 24 h. The mRNA expression levels of *VDAC1, MCU1, MICU1, MCUR1*, and *MCUB* were quantified by qPCR analysis. N = 5. **C** Cells were treated with C3A for 30 min and exposed to DHT for 24 h. Protein expression levels of MCU1 and VDAC1 were analyzed by western blotting. N = 4. **D** Interaction between VDAC1 and IP3R1 (VDAC1–IP3R1, red) in DPC cells was assessed by PLA assay. N = 4. Magnification ×1000. Scale bars are 8 μm. **E**, **F** Cells were pretreated with RU360 (1 μM) for 30 min and exposed to DHT for 72 h. **E** SA-β-gal activity assay was performed, and blue stained cells of total cells were counted. N = 5. **F** Protein expression levels of p16 and p21 were quantified by western blot analysis. N = 4. *p < 0.05 versus Control. ^#^p < 0.05 versus DHT

MAM enhances communication between the mitochondria and ER, and it plays a role in transfer of calcium into mitochondria and the aging process. Here, we investigated the effect of C3A on DHT-induced MAM formation. DHT increased the colocalization of the ER-Tracker and Mito-Tracker in DPCs, which was inhibited by C3A pretreatment (Fig. 4A). Then, we investigated the effect of DHT on mRNA expression of mitochondrial calcium influx regulatory proteins such as VDAC1, mitochondrial calcium uniporter (MCU), mitochondrial calcium uptake 1 (MICU1), MCU regulator 1 (MCUR1), and MCU-dominant negative beta subunit (MCUB). We found that mRNA expression level of *VDAC1* was upregulated, whereas no significant change was detected in the mRNA levels of *MCU*, *MICU1*, *MCUR1*, or *MCUB* under DHT treatment in DPCs (Fig. 4B). In contrast, C3A pretreatment reduced the DHT-induced VDAC1 expression (Fig. 4C). To confirm whether MAM formation is increased due to VDAC1 upregulation by DHT, we performed PLA to detect the connection between IP3R1 and VDAC1. We found that increased interaction between VDAC1 and IR3R1 was promoted by DHT treatment, and it was reduced with C3A pretreatment (Fig. 4D). Inhibition of DHT-induced mitochondrial calcium accumulation by MCU inhibitor, RU360, decreased the SA-β-gal-positive cell population and senescence marker p16 and p21 protein expression (Fig. 4E and F). Based on these results, our data suggests that DPC senescence is reduced by downregulation of VDAC1 and C3A reduces ER-mitochondria contact by inhibition of VDAC1–IP3R1 interaction.

### Effects of C3A on DHT-induced mitochondrial calcium accumulation

**Fig. 5:**
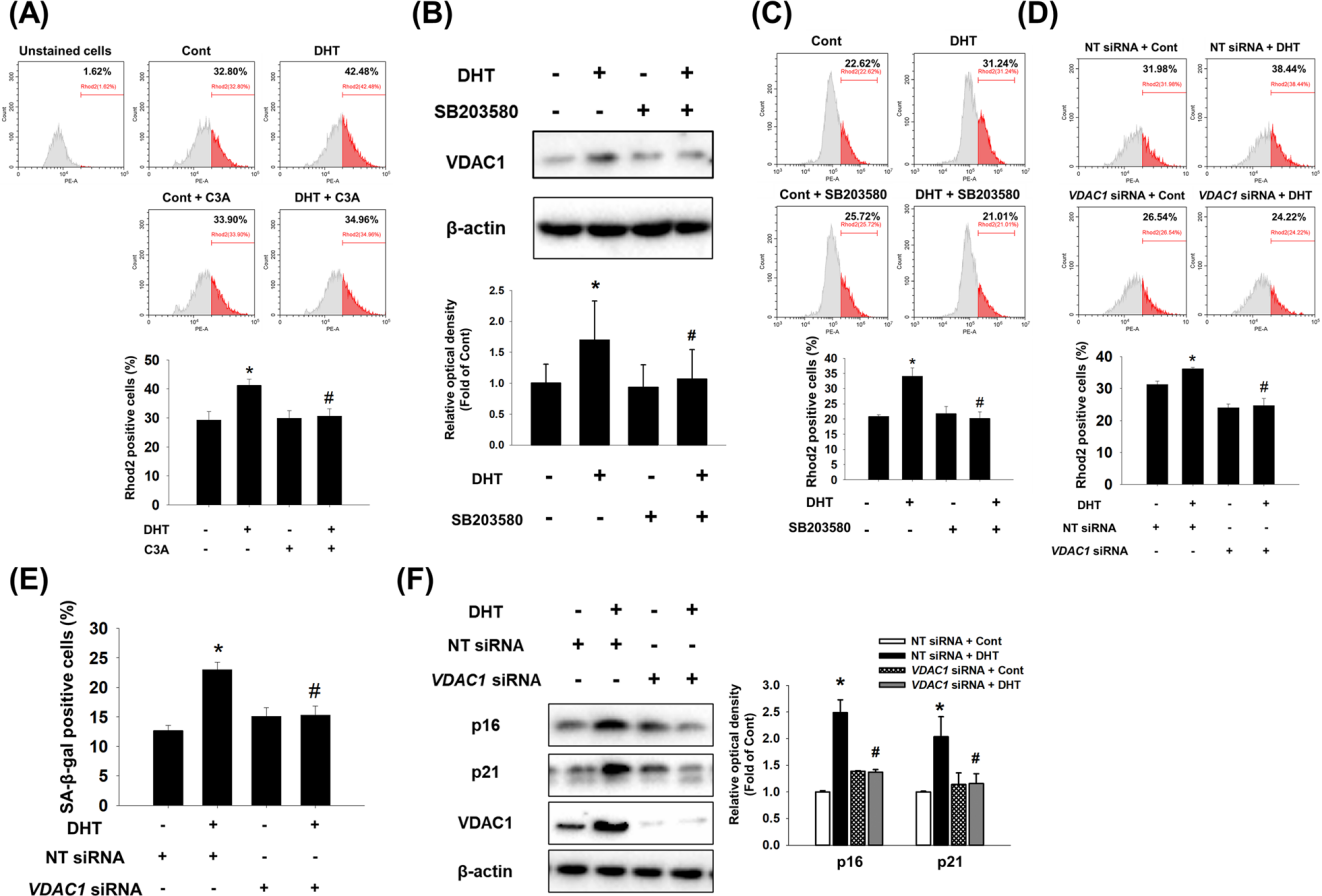
Effect of C3A on DHT-induced mitochondrial calcium accumulation. **A** DPCs were treated with C3A for 30 min and exposed to DHT for 48 h. Cells were stained with Rhod-2 (2 μM) and positive cells were analyzed with flow cytometer. N = 6. **B** Cells were pretreated with SB203580 (1 μM) for 30 min and exposed to DHT for 24 h. VDAC1 expression level was analyzed by western blotting. **C** Cells were pretreated with SB203580 for 30 min and exposed to DHT for 48 h. Flow cytometric analysis was performed with Rhod-2 (2 μM) staining and phycoerythrin (PE)-positive cells were quantified. **D** Cells were transfected with NT siRNA (25 nM) and *VDAC1* siRNA (25 nM) and exposed to DHT for 48 h. Flow cytometric analysis was achieved with Rhod-2 staining and PE-positive cells were quantified. N = 4. **E**, **F** Silencing with *VDAC1* siRNA (25 nM) or NT siRNA (25 nM) was done and the cells were exposed to DHT for 72 h. **E** SA-β-gal activity assay was performed, and blue stained cells of total cells were counted. N = 5. **F** Protein expression levels of p16, p21 and VDAC1 were quantified by western blot analysis. N = 4. *p < 0.05 versus Control. ^#^p < 0.05 versus DHT or NT siRNA + DHT

Mitochondrial calcium accumulation leads to mitochondrial dysfunction, which induces cellular senescence [31]. Thus, we measured mitochondrial calcium influx by staining Rhod-2. The results from detecting Rhod-2 fluorescence by flow cytometry showed that C3A pretreatment inhibited DHT-stimulated mitochondrial calcium influx in DPC (Fig. 5A). We further confirmed that inhibition of p38 reversed DHT-induced VDAC1 expression and mitochondrial calcium accumulation (Fig. 5B and C). Silencing of AR did not suppress the DHT-induced VDAC1 expression (Additional file 1: Fig. S4). We then investigated whether the inhibition of mitochondrial calcium accumulation induces anti-aging effects through suppression of VDAC1 expression in DPCs. Silencing VDAC1 reduced the DHT-stimulated Rhod-2-positive cell population (Fig. 5D). Transfection of *VDAC1* siRNA decreased the SA-β-gal-positive cell population and expression of p21 and p16 under DHT treatment (Fig. 5E and F). These findings indicate that C3A suppresses DHT-induced mitochondrial calcium accumulation via regulation of p38-mediated VDAC1 expression, and excessive mitochondrial calcium is involved in inducing DPC senescence.

### Effect of C3A on hair growth cycles in an AGA mice model

**Fig. 6:**
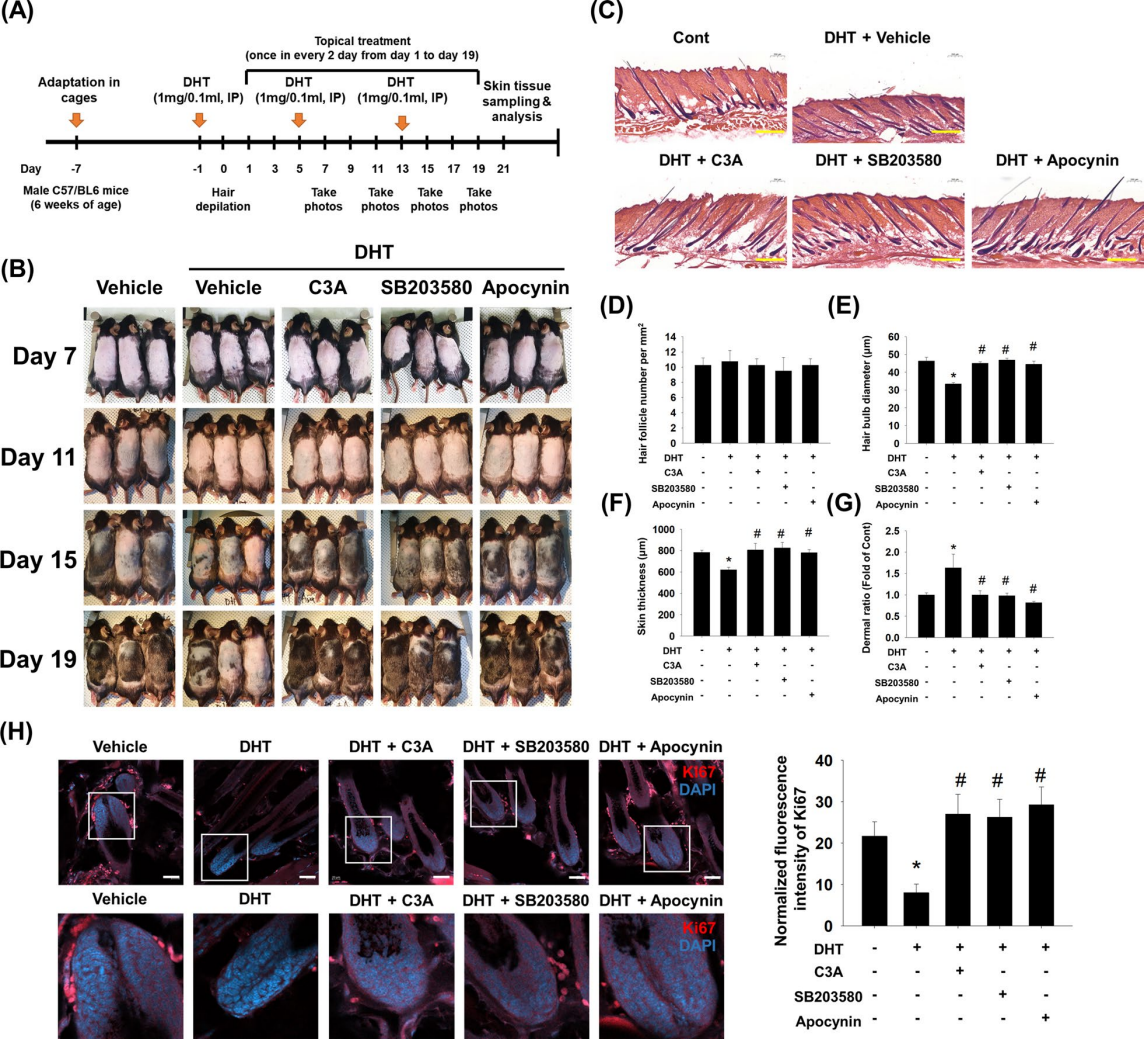
Effect of C3A on hair growth cycles in an AGA mice model. **A** Animal experimental schedule for inducing androgenetic alopecia mice model. DHT (1 mg/100 μl) was intraperitoneally injected and C3A (500 μM), SB203580 (1 mM), and Apocynin (100 mM) was topically administered once every 2 days. **B** Photographs were taken on days 7, 11, 15 and 19 after depilation. **C** Depilated mice skin tissue was frozen sectioned and histologic examination was performed by HE staining. Images were captured by digital slide scanner. N = 6. Scale bars are 400 μm. **D** Hair follicle number was counted in the same random area (1 mm^2^). **E** Hair bulb diameter was measured. **F** Skin thickness was assessed. **G** Dermal ratio was rated by measuring epidermis and dermis length of skin tissue section. **H** Immunofluorescence staining of Ki67 in the dorsal skin was performed with anti-Ki67 antibody and DAPI for nuclei staining. Magnification × 200. Scale bars are 40 μm. *p < 0.05 versus Control. ^#^p < 0.05 versus DHT + Vehicle

DHT-induced DPC senescence is associated with hair growth deceleration and changes in HFSCs, which are inhibited by C3A-induced p38 phosphorylation and MAM formation. We investigated the effects of C3A on hair regrowth induction in a DHT-induced alopecia mice model. To examine the effect of C3A-mediated p38 phosphorylation via NOX dependent ROS formation on DHT-induced hair regrowth reduction, mice were treated with DHT via intraperitoneal injection. C3A, SB203580 and Apocynin were topically administered on the hairless skin as shown in Fig. 6A. In gross examination results from pictures taken at days 7, 11, 15, and 19 after hair depilation, C3A rescued the DHT-induced hair growth deceleration (Fig. 6B). In addition, SB203580 and Apocynin results showed that hair growth rate was accelerated compared to the vehicle-administered mice group. At day 21 after treatment, skin tissue samples were acquired, and tissue slides were stained with HE (Fig. 6C). DPCs are located in the hair bulb of HFs and are associated with the progress of hair growth and the cycles of HFs. Since hair follicle numbers and hair bulb size could be modulated by AGA pathogenesis, we observed the effect of DHT on them. The number of hair follicles is not decreased in mice treated with DHT, but hair bulb size was significantly reduced by DHT and C3A, SB203580, and Apocynin could reverse the effect of DHT (Fig. 6D and E). This suggests that C3A and C3A-mediated inhibition of p38 phosphorylation is involved in maintaining the DPC proliferation and function despite the exposure to DHT. DHT also alters the thickness of the skin layer. We examined the skin thickness and dermis to epidermis ratio. The skin thickness was also reduced in DHT-injected mice, but C3A could reverse this thinning of the skin (Fig. 6F and G). Mouse skin tissue sections were stained with an anti-Ki67 antibody, and immunofluorescent signals in hair follicle regions were evaluated at day 21. The number of Ki67-positive cells was decreased in the tissue section acquired from DHT-induced mice, but C3A treatment increased the number of Ki67-positive signals. This indicates that C3A could induce anagen phase from telogen phase (Fig. 6H). Taken together, DHT-induced hair follicle cycle delay and hair growth deceleration were regulated by C3A and p38- and NOX-mediated signaling. Also, the recovery of hair growth rate by C3A is associated with inhibition of DPC senescence.

## Discussion

In this study, we show the effect of C3A on p38-mediated MAM formation and mitochondrial calcium influx, which alleviates DHT-induced DPC senescence. Senescent cells are viable, but non-proliferative cells that are damaged from disruption of the genome and proteome integrity mostly attributed to mitochondrial dysfunction associated with mtROS accumulation [32]. They exhibit changes of DNA integrity, redox signaling, senescence associated with secretory phenotypes, and metabolic profiles [33]. In a chemical analysis of *Aronia melanocarpa* extracts, C3A showed higher radical scavenging activity against oxidative stress than other forms of cyanidins. This suggests that the scavenging capability of cyanidins is determined by the residue of linked sugar units in alleviating oxidative stress [34]. Based on previous results, including ours, C3A, a cyanidin conjugated with an arabinoside sugar in carbon 3 position, has a higher antioxidative efficiency than other cyanidins [35]. The protective effects of other cyanidins against oxidative stress-induced senescence through maintenance of mitochondrial membrane potential and mitochondrial functions have been previously reported [23, 24, 36]. But, our results revealed that C3A reversed DHT-induced mtROS levels and senescence marker expression in DPCs. Therefore, we found that C3A plays a role in reducing DHT-induced DPC senescence and regulating mitochondrial functions.

Our study shows that inhibition of AR nuclear translocation is not sufficient for suppressing DHT-induced DPC senescence. Although DHT stimulates the secretion of inhibitory factors, such as DKK-1, TGF-β, and IL-6, it is not involved in the process of DPC senescence. A previous report shows that DHT induces signal transduction stimulating DPC senescence, which is regulated regardless of nuclear translocation of AR [37]. Therefore, mAR signaling-mediated DPC senescence can be a target for AGA pathogenesis. In our study, we identified a strong anthocyanin compound, C3A, that has effects on both intracellular and mAR signaling in the process of DPC senescence. A previous report has shown that androgens induce oxidative stress via activation of mAR lacking its regulatory N-terminal domain variant, which induces NOX-dependent ROS generation [30]. Also, DHT facilitates signal transduction via activation of putative mARs such as SLC39A, GPRC6A, and transient receptor potential melastatin 8 (TRPM8) [38]. Consistent with our results, other researchers have shown that androgens have distinctive effects dependent on oxidative stress levels, but AR inhibition by antagonists could not rescue the oxidative stress-induced neuronal cell viability [30]. Conversely, nuclear translocation of AR induces an adaptation mechanism and androgen deprivation provokes oxidative damage with an increase of NOXs (NOX1, gp91^phoz^, and NOX4) and a decrease of ROS reducing enzymes. This suggests that nuclear translocation of AR regulates ROS balance against oxidative stress [39–41]. Therefore, DHT-induced senescence is not dependent on nuclear translocation of AR. But, it suggests that the antioxidative effects of C3A are crucial for recovering DPC senescence by scavenging ROS produced by mAR-mediated signaling.

Our study initially demonstrated the DHT-induced DPC senescence is facilitated by p38 phosphorylation and C3A inhibits mAR signaling-mediated p38 phosphorylation. In our results, DPC senescence is induced by p38-mediated mitochondrial dysfunction and VDAC1 expression is increased by DHT. Consistent with our results, in silico analysis suggested that the *VDAC1* promoter sequence is predicted to bind with the CREB, SP1, and ETSF [42]. These transcription factors are regulated by p38 kinase activation [43]. It is supposed that VDAC1 expression is regulated by p38-mediated causative transcription factors binding with the *VDAC1* promoter sequence. Although the VDAC1 expression regulatory mechanism remains unknown, these findings suggest the possibility that VDAC1 plays a crucial role in ER-mitochondria contacts by assembling the VDAC1–IP3R1 complex. Mitochondrial calcium uptake is known to be accelerated by MAM formation, transferring calcium from ER to mitochondria by IP3R1–VDAC1 coupling. Excessive calcium influx consequently overactivates citrate cycle enzymes and ATP synthase, leading to mtROS accumulation and cellular senescence [15]. Previous reports show that intracellular calcium levels are increased in the process of HFSC differentiation by mitochondrial ATP synthase activation and keratinocyte differentiation is regulated by cytosolic calcium level [44, 45]. To the best of our knowledge, there has been no study on the effect of MAM formation and mitochondrial calcium accumulation on DPC senescence. Significantly, mAR-mediated signaling induces DPC senescence with mitochondrial calcium accumulation. Furthermore, this study is the first to report that C3A acts as a regulator of the VDAC1–IP3R1 complex via p38-mediated VDAC1 expression. Our results also show that MCU expression is not altered by DHT but MCU inhibition reverses DHT-induced DPC senescence. These results imply that MCU expression does not determine mitochondrial calcium entry level but acts as a passive transporter of calcium into the mitochondrial matrix. Previous studies support these results that MCU-mediated mitochondrial calcium overload is involved in aging-related epigenetic modification and MCU inhibition suppressed high glucose-induced mitochondrial dysfunction and neuronal cell aging [13, 46–48]. Since C3A inhibits DHT-induced ER-mitochondria contacts in a p38-dependent manner, it can be a promising candidate for AGA drugs regulating the influx of mitochondrial calcium to reduce DPC senescence.

Since DPC senescence is a hallmark of AGA with secretion of hair follicle inhibitory factors, we utilized the DHT-induced AGA mice model to examine whether C3A can recover DHT-induced hair cycle retention. DHT injected into the body does not directly affect HFSC proliferation, but DHT-induced senescence of DPCs in dermal papilla leads to suppression of hair follicle regeneration [49]. Miniaturization of hair follicles is engaged in the AGA process by the increased ratio of DHT to testosterone. In histological examination, our results show that DHT-treated mice with vehicle treatment have shrunken bulb sizes, but DHT-treated mice with C3A treatment show similar bulb size to control mice not treated with DHT. DHT-induced hair growth cycle deceleration was also restored by inhibition of NOX- and p38-mediated signaling by treatment of Apocynin or SB203580. These results show that C3A-mediated effects of antioxidants and p38 signaling inhibition are involved in slowing AGA pathogenesis. A previous report demonstrates that hair follicle cycle regulation is associated with mitochondrial dysfunction in mtDNA-depleter mice models with alteration of senescence-associated gene expressions [49]. In addition, our results show that C3A reduced inhibitory factors such as DKK-1, TGF-β1, and IL-6 produced by senescent DPCs. These inhibitory factors hindered the transition of telogen to anagen, which consequently contribute to hair follicle cycle deceleration [50]. There have been many trials for developing drugs for AGA with fewer side effects such as prostaglandins and their antagonists, Wnt signaling activators, and stem cell therapy [51]. However, our study solidifies the involvement of mitochondrial calcium in DHT-induced DPC senescence and the mAR-mediated signaling, which will be a target for novel drug development in AGA prevention. Therefore, C3A-mediated anti-senescent effects will improve the DHT-induced hair follicle cycle delay caused by mitochondrial dysfunction of DPCs and increased expression of senescence-associated hair follicle cycle inhibitory factors.

## Conclusions

**Fig. 7:**
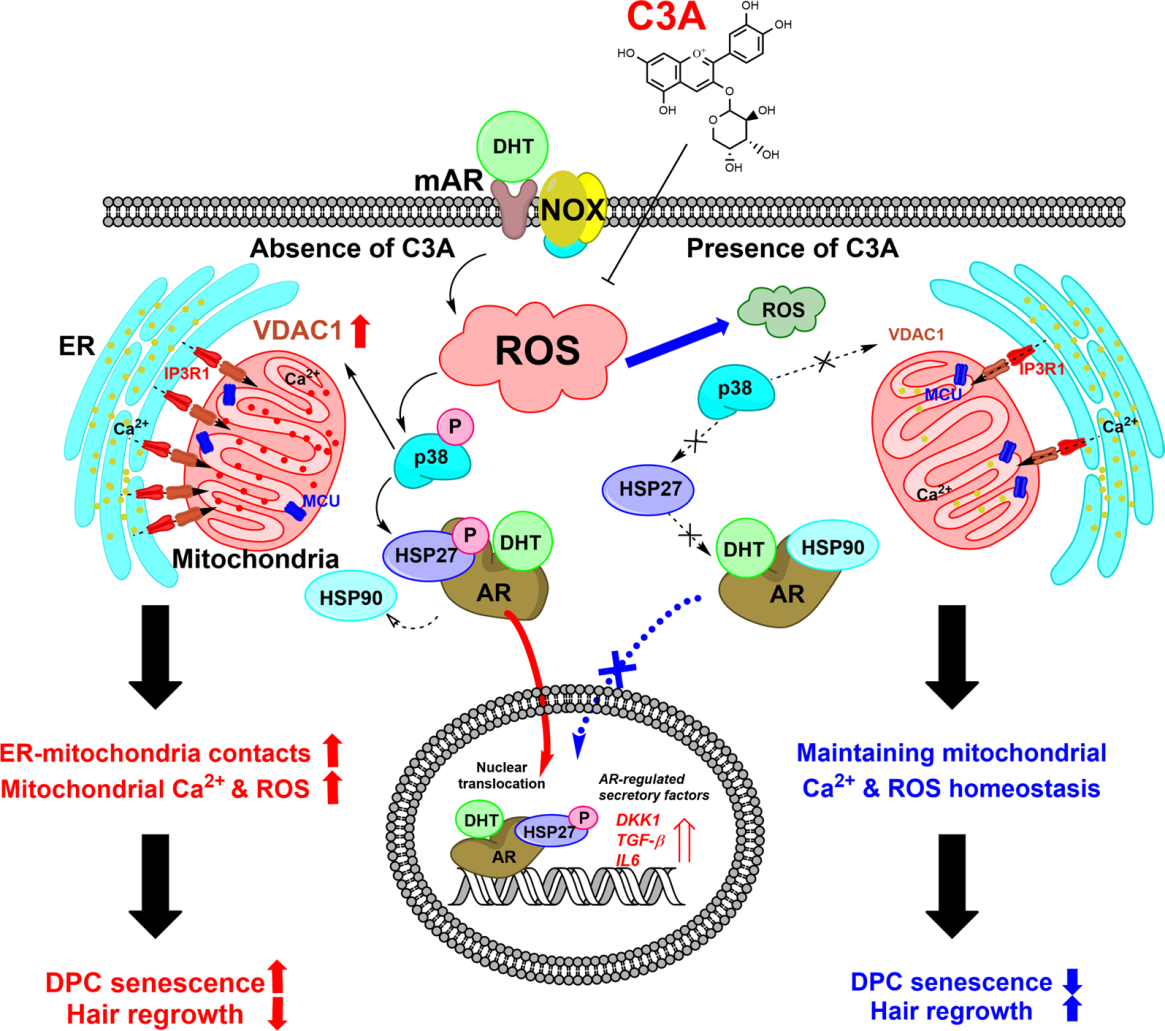
Schematic model of effect of C3A on DHT-induced mitochondrial calcium influx and DPC senescence. C3A effectively decreased DHT-induced mtROS accumulation and DPC senescence. HSP27 phosphorylation was modulated by p38-mediated signaling and it was involved in AR nuclear translocation. In turn, HSP27 phosphorylation is modulated by membrane AR-mediated signaling, which was inhibited by C3A. Remarkably, C3A inhibited p38-mediated VDAC1 expression that contributes to MAM formation and transfer of calcium via VDAC1–IP3R1 interactions. Finally, excessive mitochondrial calcium entry through MAM formation resulted in increase of DPC senescence, but C3A reduced the mitochondrial calcium influx under DHT exposure conditions

The scavenge effect of C3A prevents ROS signaling caused by the NOX complex, and p38-mediated signaling is a crucial promoter of DHT-induced DPC senescence through MAM formation and mitochondrial dysfunction (Fig. 7). Therefore, our study suggests that C3A is a promising natural organic compound with incredible potential for AGA treatment or supportive medication against DHT-induced DPC senescence.

## Supplementary Information

**Additional file 1: Figure S1.** Effect of C3A in DHT-induced IL-6 and TGF-β1 release.** Figure S2. **Effect of C3A on p38-mediated signaling pathway to reverse DPC senescence.** Figure S3. **Expression of membrane ARs and the effect of *AR* siRNA on membrane AR expressions in DPCs. **Figure S4.** Effect of silencing AR in VDAC1 expression. **Table S1. **Array map of human growth factor antibody array C1. **Table S2. **Sequences of primers used for real-time PCR and siRNA.

## Data Availability

Not applicable.

## Abbreviations

AGA: Androgenetic alopecia
DHT: Dihydrotestosterone
DPCs: Dermal papilla cells
C3A: Cyanidin 3-*O*-arabinoside
AR: Androgen receptor
VDAC1: Voltage-dependent anion channel 1
MAM: Mitochondria-associated ER membrane
mtROS: Mitochondrial ROS
HSP27: Heat shock protein 27
NOX: NADPH oxidase
MAPK: Mitogen-activated protein kinase
MCU: Mitochondrial calcium uniporter
MICU1: Mitochondrial calcium uptake 1
MCUR1: MCU regulator 1
MCUB: MCU-dominant negative beta subunit
RT-PCR: Reverse-transcription PCR
