# Supplementary material for: Cyanidin 3-O-arabinoside suppresses DHT-induced dermal papilla cell senescence by modulating p38-dependent ER-mitochondria contacts
Source: J Biomed Sci. 2022 Mar 7;29:17. doi: 10.1186/s12929-022-00800-7 (PMC8900350; doi:10.1186/s12929-022-00800-7)
Supplement: Supplementary file 1 — Additional file 1: Figure S1. Effect of C3A in DHT-induced IL-6 and TGF-β1 release. Figure S2. Effect of C3A on p38-mediated signaling pathway to reverse DPC senescence. Figure S3. Expression of membrane ARs and the effect of AR siRNA on membrane AR expressions in DPCs. Figure S4. Effect of silencing AR in VDAC1 expression. Table S1. Array map of human growth factor antibody array C1. Table S2. Sequences of primers used for real-time PCR and siRNA. [file 12929_2022_800_MOESM1_ESM.docx]

**Cyanidin 3-O-Arabinoside Suppresses DHT-Induced Dermal Papilla Cell Senescence by Modulating p38-Dependent ER-Mitochondria Contacts**

Young Hyun Jung^1, a^, Chang Woo Chae^1, a^, Gee Euhn Choi^1, a^, Him Cha Shin^4^, Jae Ryong Lim^1^, Han Seung Chang^1^, Joonmo Park^1^, Ji Hyeon Cho^1^, Mo Ran Park^1^, Hyun Jik Lee^2, 3^, and Ho Jae Han^1, *^

**Affiliations**

^1^Department of Veterinary Physiology, College of Veterinary Medicine, Research Institute for Veterinary Science, and BK21 PLUS Program for Creative Veterinary Science Research, Seoul National University, Seoul, 08826, South Korea

^2^Laboratory of Veterinary Physiology, College of Veterinary Medicine, Chungbuk National University, Cheongju, Chungbuk, 28644, South Korea

^3^Institute for Stem Cell & Regenerative Medicine (ISCRM), Chungbuk National University, Cheongju, Chungbuk, 28644, South Korea

^4^Biomedical Research Institute, Stempoint Co., Ltd. Seoul, 08501, South Korea

^a^These authors contributed equally to this work

**^*^**Corresponing author: Ho Jae Han, D.V.M., Ph.D.

Department of Veterinary Physiology, College of Veterinary Medicine and Research Institute for Veterinary Science, Seoul National University, Seoul 08826, Republic of Korea

Tel: +82-2-880-1261

Fax: +82-2-880-2732

E-mail: hjhan@snu.ac.kr

Running title: Effect of C3A on DHT-induced DPC senescence

**
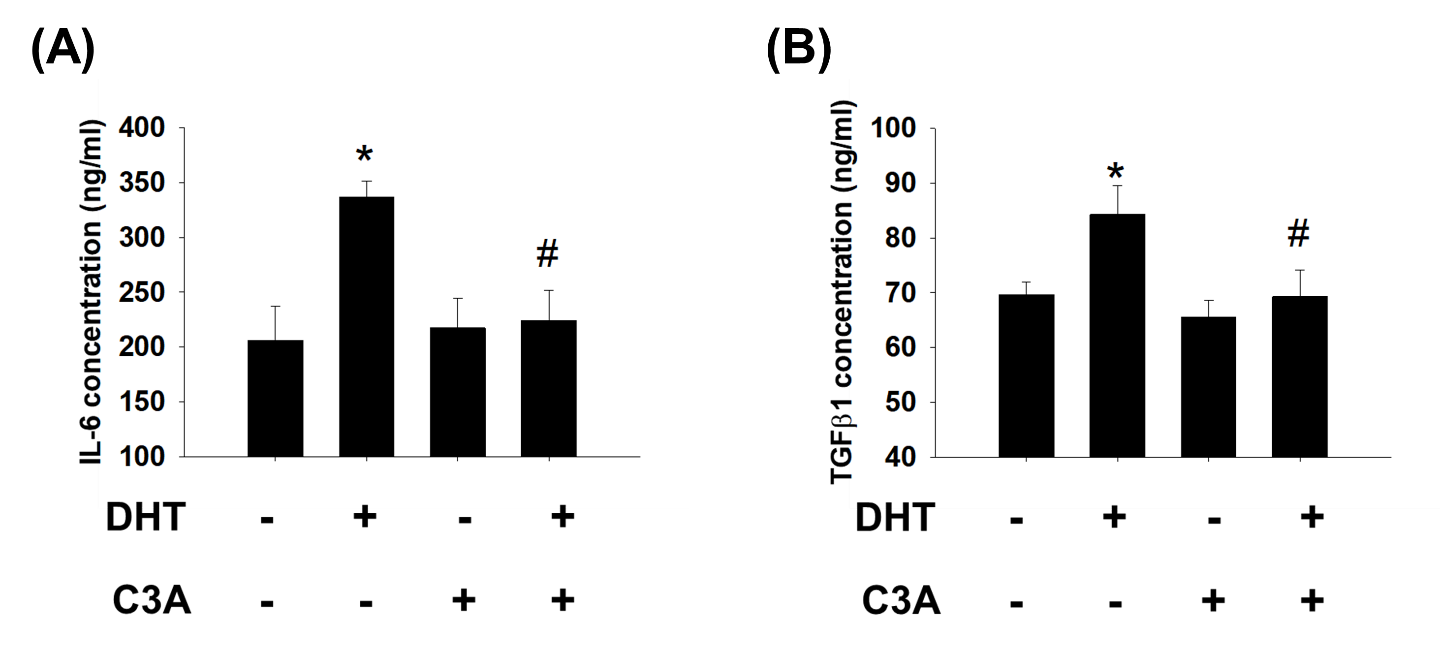
**

**Supplementary Figure 1.** **Effect of C3A in DHT-induced IL-6 and TGF-β1 release.** (A and B) IL-6 and TGF-β1 concentrations in the DPC conditioned media by using ELISA kits. DPC conditioned media was taken from the culture media of DPCs pretreated with C3A and treated with DHT for 72 h. N = 5. Data are mean±SEM. *p <0.05 versus Control. #p <0.05 versus DHT.

**
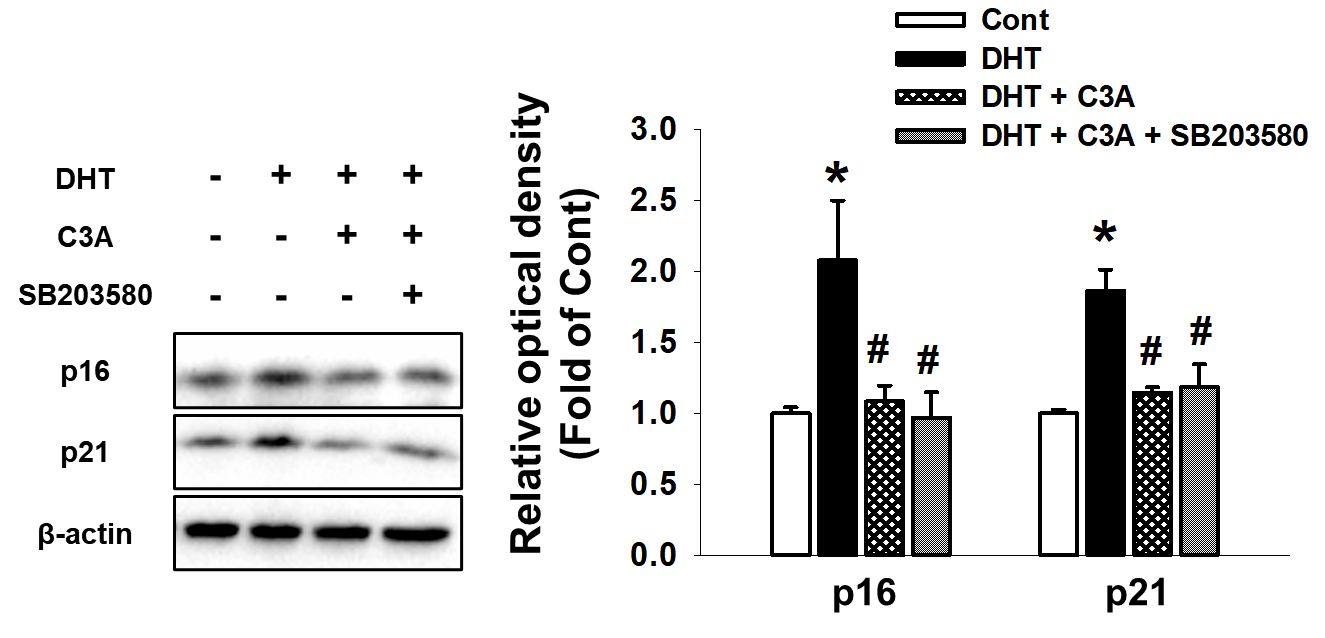
**

**Supplementary Figure 2. Effect of C3A on p38-mediated signaling pathway to reverse DPC senescence.** DPCs were treated with C3A (1 μM) and SB203580 (1 μM) for 30 min and exposed to DHT for 72 h. Protein expression levels of p16 and p21 were quantified by western blot analysis. N = 4. **P* < 0.05 vs. control, ^#^*p* < 0.05 vs. DHT.

**
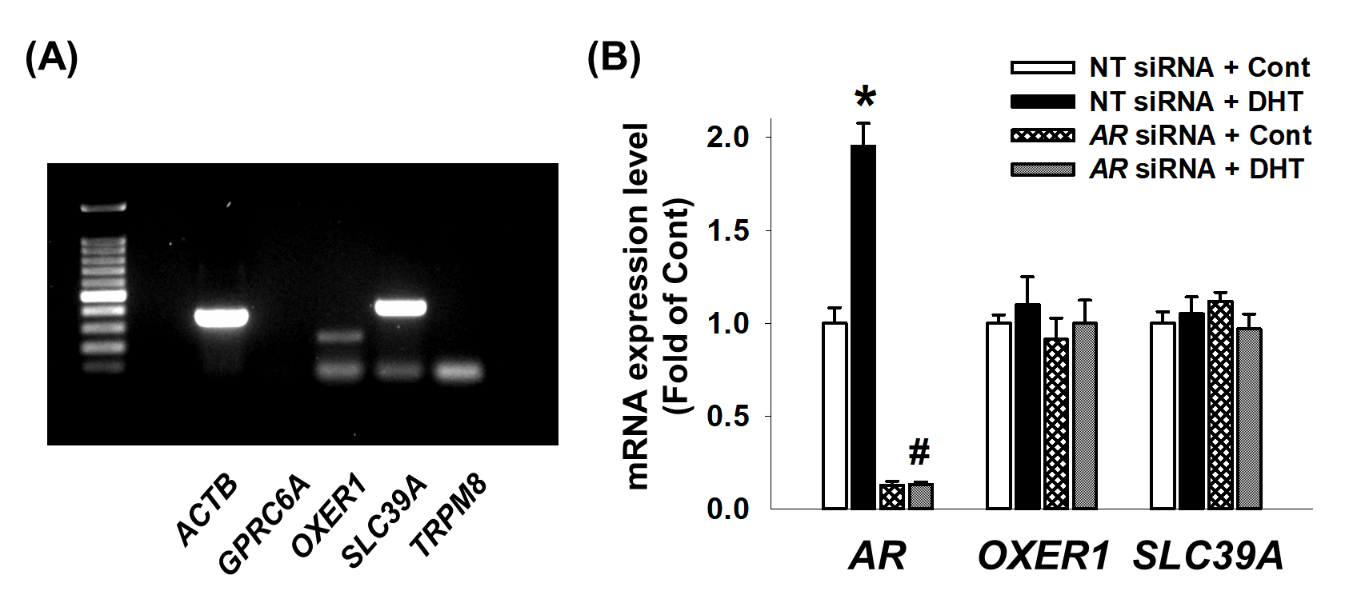
**

**Supplementary Figure 3. Expression of membrane ARs and the effect of *AR* siRNA on membrane AR expressions in DPCs.** (A) The mRNA expression levels of *GPRC6A*, *OXER1*, *SLC39A* and *TRPM8* in DPCs were examined with PCR analysis. cDNA was synthesized with 500 ng of RNA and PCR was performed with 40-cycle reactions. Primers used for PCR reactions are listed in Supplementary Table 2. (B) DPCs were transfected with NT siRNA (25 nM) or *AR* siRNA (25 nM) and treated with DHT (100 nM) for 24 h. The mRNA expression levels of *AR*, *OXER1*, and *SLC39A* were analyzed by real time PCR. **P* < 0.05 vs. NT siRNA, ^#^*p* < 0.05 vs. NT siRNA + DHT.

**
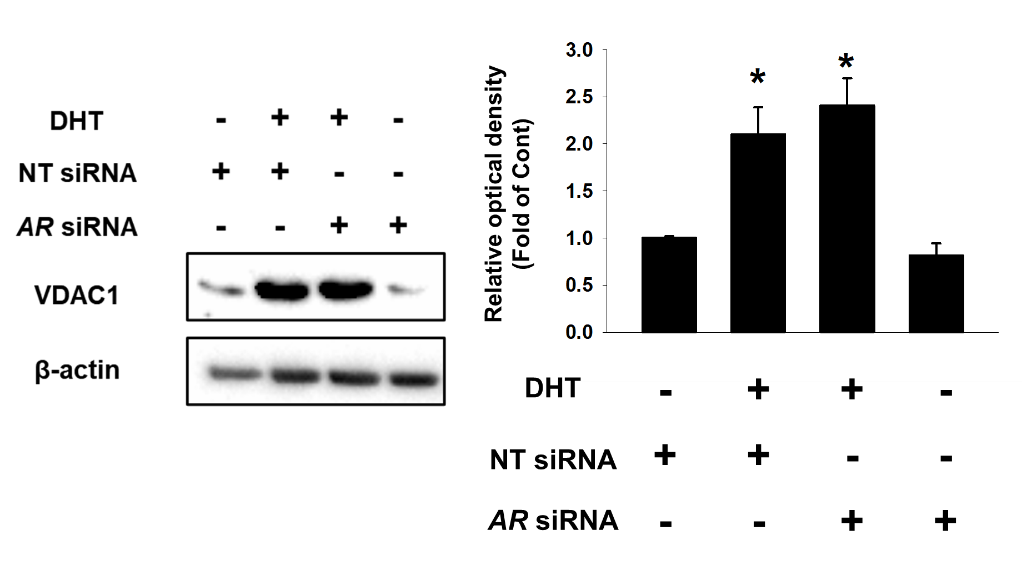
**

**Supplementary Figure 4.** **Effect of silencing AR in VDAC1 expression.** Dermal papilla cells were transfected with NT siRNA (25 nM) or *AR* siRNA (25 nM) for 24 h and treated with DHT (100 nM) for 24 h. VDAC1 expression level was analyzed by western blotting. β-actin was used as a loading control. N = 3. Data are mean±SEM. *p <0.05 versus Control. #p <0.05 versus NT siRNA + DHT.

**Supplementary Table 1. Array map of human growth factor antibody array C1**

|  | **A** | **B** | **C** | **D** | **E** | **F** | **G** | **H** | **I** | **J** | **K** | **L** |
| --- | --- | --- | --- | --- | --- | --- | --- | --- | --- | --- | --- | --- |
| **1** | POS | POS | NEG | NEG | [AR](http://www.copewithcytokines.de/cope.cgi?680) | [bFGF](http://www.copewithcytokines.de/cope.cgi?1125) | [bNGF](http://www.copewithcytokines.de/cope.cgi?7074) | [EGF](http://www.copewithcytokines.de/cope.cgi?3170) | EGF R | [FGF-4](http://www.copewithcytokines.de/cope.cgi?3606) | [FGF-6](http://www.copewithcytokines.de/cope.cgi?3609) | [FGF-7](http://www.copewithcytokines.de/cope.cgi?3610) |
| **2** | POS | POS | NEG | NEG | [AR](http://www.copewithcytokines.de/cope.cgi?680) | [bFGF](http://www.copewithcytokines.de/cope.cgi?1125) | [bNGF](http://www.copewithcytokines.de/cope.cgi?7074) | [EGF](http://www.copewithcytokines.de/cope.cgi?3170) | EGF R | [FGF-4](http://www.copewithcytokines.de/cope.cgi?3606) | [FGF-6](http://www.copewithcytokines.de/cope.cgi?3609) | [FGF-7](http://www.copewithcytokines.de/cope.cgi?3610) |
| **3** | [G-CSF](http://www.copewithcytokines.de/cope.cgi?3609) | [GDNF](http://www.copewithcytokines.de/cope.cgi?5125) | [GM-CSF](http://www.copewithcytokines.de/cope.cgi?4962) | [HB-EGF](http://www.copewithcytokines.de/cope.cgi?8301) | [HGF](http://www.copewithcytokines.de/cope.cgi?7212) | [IGFBP-1](http://www.copewithcytokines.de/cope.cgi?7211) | [IGFBP-2](http://www.copewithcytokines.de/cope.cgi?3985) | [IGFBP-3](http://www.copewithcytokines.de/cope.cgi?4140) | [IGFBP-4](http://www.copewithcytokines.de/cope.cgi?4966) | [IGFBP-6](http://www.copewithcytokines.de/cope.cgi?4967) | [IGF-I](http://www.copewithcytokines.de/cope.cgi?5123) | IGF-I SR |
| **4** | [G-CSF](http://www.copewithcytokines.de/cope.cgi?3985) | [GDNF](http://www.copewithcytokines.de/cope.cgi?4009) | [GM-CSF](http://www.copewithcytokines.de/cope.cgi?4140) | [HB-EGF](http://www.copewithcytokines.de/cope.cgi?4398) | [HGF](http://www.copewithcytokines.de/cope.cgi?4590) | [IGFBP-1](http://www.copewithcytokines.de/cope.cgi?4965) | [IGFBP-2](http://www.copewithcytokines.de/cope.cgi?4966) | [IGFBP-3](http://www.copewithcytokines.de/cope.cgi?4967) | [IGFBP-4](http://www.copewithcytokines.de/cope.cgi?4968) | [IGFBP-6](http://www.copewithcytokines.de/cope.cgi?4970) | [IGF-I](http://www.copewithcytokines.de/cope.cgi?4962) | IGF-I SR |
| **5** | [IGF-II](http://www.copewithcytokines.de/cope.cgi?4963) | [M-CSF](http://www.copewithcytokines.de/cope.cgi?6276) | MCSF R | [NT-3](http://www.copewithcytokines.de/cope.cgi?7211) | [NT-4](http://www.copewithcytokines.de/cope.cgi?7212) | PDGF Ra | [PDGF Rb](http://www.copewithcytokines.de/cope.cgi?7539) | [PDGF-AA](http://www.copewithcytokines.de/cope.cgi?7531) | [PDGF-AB](http://www.copewithcytokines.de/cope.cgi?7532) | [PDGF-BB](http://www.copewithcytokines.de/cope.cgi?7535) | PIGF | [SCF](http://www.copewithcytokines.de/cope.cgi?8301) |
| **6** | [IGF-II](http://www.copewithcytokines.de/cope.cgi?4963) | [M-CSF](http://www.copewithcytokines.de/cope.cgi?6276) | MCSF R | [NT-3](http://www.copewithcytokines.de/cope.cgi?7211) | [NT-4](http://www.copewithcytokines.de/cope.cgi?7212) | PDGF Ra | [PDGF Rb](http://www.copewithcytokines.de/cope.cgi?7539) | [PDGF-AA](http://www.copewithcytokines.de/cope.cgi?7531) | [PDGF-AB](http://www.copewithcytokines.de/cope.cgi?7532) | [PDGF-BB](http://www.copewithcytokines.de/cope.cgi?7535) | PIGF | [SCF](http://www.copewithcytokines.de/cope.cgi?8301) |
| **7** | SCF R | [TGF-a](http://www.copewithcytokines.de/cope.cgi?8060) | [TGF-b](http://www.copewithcytokines.de/cope.cgi?8301) | [TGF-b2](http://www.copewithcytokines.de/cope.cgi?4963) | [TGF-b3](http://www.copewithcytokines.de/cope.cgi?6276) | [VEGF](http://www.copewithcytokines.de/cope.cgi?9413) | VEGF R2 | VEGF R3 | [VEGF D](http://www.copewithcytokines.de/cope.cgi?7539) | Blank | Blank | POS |
| **8** | SCF R | [TGF-a](http://www.copewithcytokines.de/cope.cgi?9188) | [TGF-b](http://www.copewithcytokines.de/cope.cgi?9190) | [TGF-b2](http://www.copewithcytokines.de/cope.cgi?9201) | [TGF-b3](http://www.copewithcytokines.de/cope.cgi?9202) | [VEGF](http://www.copewithcytokines.de/cope.cgi?9997) | VEGF R2 | VEGF R3 | [VEGF D](http://www.copewithcytokines.de/cope.cgi?10013) | Blank | Blank | POS |

**Supplementary Table 2. Sequences of primers used for real-time PCR and siRNA**

| Gene | Identification | | Sequence (5'-3') |
| --- | --- | --- | --- |
| *AR* | Sense | | CCTGGCTTCCGCAACTTACAC |
|  | Antisense | | GGACTTGTGCATGCGGTACTCA |
| *GPRC6A* | Sense | | CTCCGTGCACTTAATGATTCCTA |
|  | Antisense | | AAGCCAACACACTCCTGGAT |
| *OXER1* | Sense | | TGCCTCAACCAACTGGACAA |
|  | Antisense | | TGACAGTGATTGGCGTCTGT |
| *SLC39A* | Sense | | CAACTTTGCTGCTGAACCCC |
|  | Antisense | | CCGGCAACAGACCCTTTTTG |
| *TRPM8* | Sense | | CAGCAGGATCCTTGGGTGAA |
|  | Antisense | | TCTGGGCATAGCCACACTTG |
| *DKK1* | Sense | | TCACGCTATGTGCTGCCCCG |
|  | Antisense | | TCTGGAATACCCATCCAAGGTGCT |
| *TGFB1* | Sense | | GGCCAGATCCTGTCCAAGC |
|  | Antisense | | GTGGGTTTCCACCATTAGCAC |
| *IL6* | Sense | | ACTCACCTCTTCAGAACGAATTG |
|  | Antisense | | CCATCTTTGGAAGGTTCAGGTTG |
| *VDAC1* | Sense | | GGAAGGCAGAAGATGGCTGTG |
|  | Antisense | | GTCACTTTGGTGGTCTCAGTGT |
| *MCU* | Sense | | GGTCCAGCAACTATACACCACA |
|  | Antisense | | CAAAGTGGTCCTCTTCTCAGCT |
| *MICU1* | Sense | | GGCTAGGAGAGTCACGTGAGAG |
|  | Antisense | | GGTACCATCGAGAACCCACAG |
| *MCUR1* | Sense | | CAGCAAGATCGGGCCCTTAC |
|  | Antisense | | TCCACAGGCGATAAAATCCCAG |
| *MCUB* | Sense | | TGACCACCTGAAGGAACAGC |
|  | Antisense | | GAGTACACCCACCACGTGAG |
| *ACTB* | Sense | | AACCGCGAGAAGATGACC |
|  | Antisense | | AGCAGCCGTGGCCATCTC |
| *AR siRNA* | Sense | GAA UCU ACC CUU CAA GUA U | |
|  | Antisense | AUA CUU GAA GGG UAG AUU C | |
|  | Sense | CGU CUA CCC UGU CUC UCU A | |
|  | Antisense | UAG AGA GAC AGG GUA GAC G | |
|  | Sense | CUC UCU UCA CAG CCG AAG A | |
|  | Antisense | UCU UCG GCU GUG AAG AGA G | |
| *VDAC1 siRNA* | Sense | | CUG GAA ACC AAG UAC AGA U |
|  | Antisense | | AUC UGU ACU UGG UUU CCA G |
|  | Sense | | CAG CUA UAA GCU UGG AAG U |
|  | Antisense | | ACU UCC AAG CUU AUA GCU G |
|  | Sense | | AGU ACA GAU GGA CUG AGU A |
|  | Antisense | | UAC UCA GUC CAU CUG UAC U |
